# Effects of different sodium–glucose cotransporter 2 inhibitors in heart failure with reduced or preserved ejection fraction: a network meta-analysis

**DOI:** 10.3389/fcvm.2024.1379765

**Published:** 2024-05-23

**Authors:** Xiaohua Lan, Huijing Zhu, Yanjie Cao, Yue Hu, Xingman Fan, Kaijie Zhang, Mengdi Wu

**Affiliations:** ^1^ Graduate School of Hebei North University, Zhangjiakou, Hebei, China; ^2^ Department of Geriatrics, Air Force Medical Center, Air Force Medical University, PLA, Beijing, China; ^3^ Graduate School of China Medical University, Shenyang, Liaoning, China

**Keywords:** sodium–glucose cotransporter 2 inhibitors, heart failure, cardiac structural remodeling, heart failure with reduced ejection fraction, heart failure with preserved ejection fraction

## Abstract

**Background:**

This systematic review and meta-analysis aimed to explore the effects of different sodium–glucose cotransporter-2 inhibitors (SGLT2i) on prognosis and cardiac structural remodeling in patients with heart failure (HF).

**Methods:**

Relevant studies published up to 20 March 2024 were retrieved from PubMed, EMBASE, Web of Science, and Cochrane Library CNKI, China Biomedical Literature Service, VIP, and WanFang databases. We included randomized controlled trials of different SGLT2i and pooled the prognosis data of patients with HF. We compared the efficacy of different SGLT2i in patients with HF and conducted a sub-analysis based on left ventricular ejection fraction (LVEF).

**Results:**

We identified 77 randomized controlled trials involving 43,561 patients. The results showed that SGLT2i significantly enhanced outcomes in HF, including a composite of hospitalizations for HF and cardiovascular death, individual hospitalizations for HF, Kansas City Cardiomyopathy Questionnaire (KCCQ) scores, left atrial volume index (LAVi), and LVEF among all HF patients (*P *< 0.05) compared to a placebo. Sotagliflozin was superior to empagliflozin [RR = 0.88, CI (0.79–0.97)] and dapagliflozin [RR = 0.86, CI (0.77–0.96)] in reducing hospitalizations for HF and CV death. Dapagliflozin significantly reduced hospitalizations [RR = 0.51, CI (0.33–0.80)], CV death [RR = 0.73, CI (0.54–0.97)], and all-cause mortality [RR = 0.69, CI (0.48–0.99)] in patients with HF with reduced ejection fraction (HFrEF). SGLT2i also plays a significant role in improving cardiac remodeling and quality of life (LVMi, LVEDV, KCQQ) (*P *< 0.05). Among patients with HF with preserved ejection fraction (HFpEF), SGLT2i significantly improved cardiac function in HFpEF patients (*P *< 0.05). In addition, canagliflozin [RR = 0.09, CI (0.01–0.86)] demonstrated greater safety compared to sotagliflozin in a composite of urinary and reproductive infections of HFpEF patients.

**Conclusion:**

Our systematic review showed that SGLT2i generally enhances the prognosis of patients with HF. Sotagliflozin demonstrated superiority over empagliflozin and dapagliflozin in a composite of hospitalization for HF and CV death in the overall HF patients. Canagliflozin exhibited greater safety compared to sotagliflozin in a composite of urinary and reproductive infections of HFpEF. Overall, the efficacy of SGLT2i was greater in HFrEF patients than in HFpEF patients.

## Introduction

Heart failure (HF) results from either contraction or relaxation dysfunction of the heart, leading to multisystem symptoms and signs. Despite a decrease in the age-standardized prevalence of HF from 1990 to 2019, the reduction is not significant, and HF remains a significant cause of disability and death worldwide (1). Currently, in developed countries (2–4), such as Britain, France, and the United States, the prevalence of HF ranges from 1.5% to 2.0%, while in developing countries (5) and regions, such as Asia and Africa, it spans from 1.3% to 6.7%. According to the latest definitions by the European and American Heart Association, HF is categorized into three main types, namely, HF with mildly reduced ejection fraction (HFrEF) (LVEF, <40%), HF with moderately reduced ejection fraction (LVEF, 40%–50%), and HF with preserved ejection fraction (HFpEF) (EF, ≥50%). However, many previous meta-analyses defined HFpEF as EF ≥ 45%, which contrasts with the current HF classification. Hence, our study adopts the definition of HFpEF ≥ 50%.

SGLT2i is a new class of antidiabetic medications originally developed for managing diabetes. Recent research demonstrated their efficacy in improving outcomes for patients with HF, such as reduction in hospitalizations, cardiovascular mortality, adverse cardiac remodeling, and other associated factors, irrespective of the presence of diabetes. In addition, adverse cardiac remodeling is a critical mechanism in the progression of HF and serves as an independent risk factor for mortality and morbidity in patients with cardiovascular disease (5). Nevertheless, the comprehensive evaluation of SGLT2i effects on adverse cardiac remodeling in HF patients remains limited, with existing studies yielding divergent results. Despite the generally favorable effects of SGLT2i on HF patients, there is a lack of consensus on the most effective SGLT2i variation for HF treatment. Therefore, we conducted a systematic review and meta-analysis of randomized controlled trials (RCT), including a subgroup analysis based on varying ejection fractions to assess the effectiveness and safety profile of six SGLT2i for HF. This study aims to offer valuable evidence to aid clinical decision-making in HF management.

## Materials and methods

### Registration

The protocol for this systematic review and meta-analysis was not registered. The data supporting this article are available in the article and its online Supplementary Material.

### Literature search

PubMed, Embase, Web of Science, Cochrane Library, CNKI, China Biomedical Literature Service, VIP, and WanFang databases were systematically searched until 20 March 2024. Additionally, the reference lists of these relevant articles were meticulously reviewed to identify any potentially overlooked trials. The search strategy employed a combination of subject words and free words. The primary search terms included “sodium–glucose cotransporter-2 inhibitors” or “SGLT2i” and “heart failure” and specific drug names such as “empagliflozin” or “dapagliflozin” or “canagliflozin” or “sotagliflozin” or “ipragliflozin” or “ertugliflozin.”

### Inclusion and exclusion criteria

#### Literature inclusion criteria

(1) Research type: RCT.
(2) Research subject: All subjects who met the current diagnostic criteria for HF. HFrEF was defined as EF < 50%, and HFpEF was defined as EF ≥ 50%.
(3) Interventions: The experimental group received SGLT2i (dapagliflozin, empagliflozin, sotagliflozin, canagliflozin, ipragliflozin, ertugliflozin), and the control group received a placebo.
(4) Outcome indicators: ① a composite of hospitalization for HF and CV death; ② hospitalization for HF; ③ CV death; ④ all-cause death; ⑤ a composite of uric and reproductive effects; ⑥ 6 min walk distance (6MWT); ⑦ NT-proBNP; ⑧ the Kansas City Cardiomyopathy Questionnaire (KCCQ); ⑨ LAVi; ⑩ E/e'; ⑪ LVMi; ⑫ LVEDV; ⑬; LVESV ⑭ LVEF; ⑮ hematocrit (HCT).

#### Literature exclusion criteria

(1) Non-RCT, (2) duplicate publication, (3) meta-analysis studies, (4) ongoing or unpublished studies, (5) studies lacking original data or where data could not be calculated, and (6) observational or cohort studies.

### Data extraction

Literature screening involved two researchers who independently reviewed articles based on the established inclusion and exclusion criteria. After individual assessments, they cross-checked their selections to ensure consistency. Key information, such as the first author's name, study design, baseline characteristics, and study endpoints, was systematically extracted from each article.

### Literature quality evaluation

The quality of the included studies was independently assessed by two researchers using the “risk of bias assessment criteria” from the Cochrane Reviewers’ Handbook (version 5.1.0). The evaluation of the RCTs involved the following components: (1) randomized method, (2) allocation concealment, (3) blinding of participant personnel and outcome assessors, (4) completeness of outcome data, (5) absence of selective outcome reporting, and (6) clarity of reasons for losses to follow-up or discontinuation.

## Statistical analysis

The statistical analysis was conducted using Stata 15.1 software for network meta-analysis. Relative risks or odds ratios were determined for dichotomous variables, while continuous variables were analyzed using the frequentist methodology in network meta-analysis. Heterogeneity was set as *I*^2^ < 50% and *p* > 0.01 for the fixed effect model. Otherwise, the random effects model was applied. Pooled results for continuous variables were expressed as the mean difference (MD). The surface under the cumulative ranking (SUCRA) was employed to indicate the preferred ranking of each treatment. Small sample effects were investigated through a network funnel plot. *P *< 0.05 was considered statistically significant.

## Results

### Basic characteristics and quality assessment

**Figure 1:**
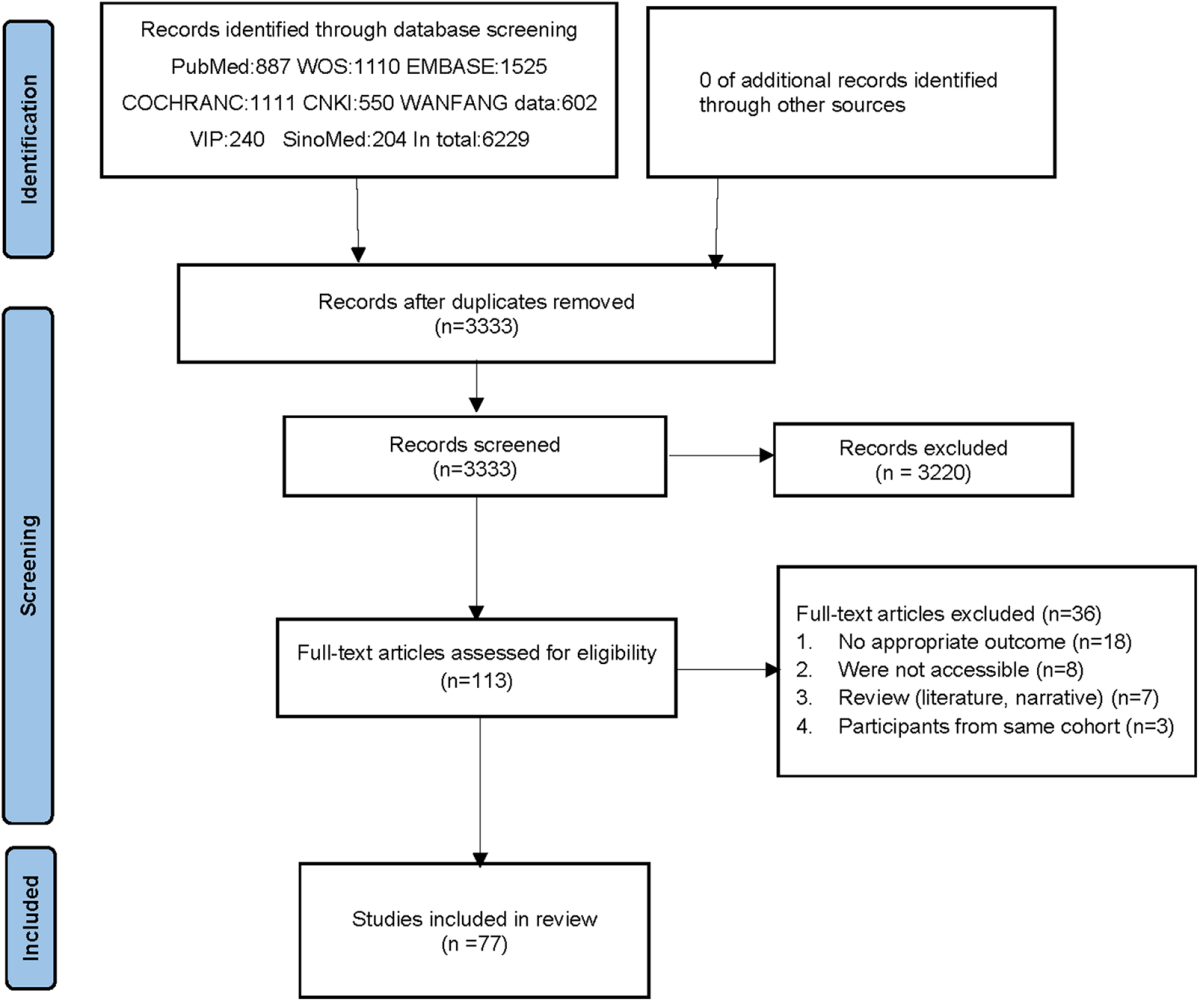
Flow diagram of study identification and selection.

A total of 6,229 relevant literature sources were identified through a comprehensive search across multiple databases. After thorough screening, 77 RCTs (6–82) were included in this study. The study encompassed a cohort of 43,561 patients diagnosed with HF, consisting of 11,734 patients with HFpEF and 31,827 patients with HFrEF. A detailed description of the literature search and screening process is illustrated in Figure 1, while the baseline characteristics are outlined in Table 1. Among the 77 selected articles, the outcome indicators related to HFpEF or HFrEF were simultaneously reported in 5 articles, 22 focused on HFpEF, and the remaining studies were centered on HFrEF. Specifically, 5 studies investigated canagliflozin treatment, which involved a total of 453 patients; 55 studies examined dapagliflozin treatment, with a collective enrollment of 16,201 patients; 16 studies utilized empagliflozin, which included 21,024 patients; 1 study explored the efficacy of ertugliflozin, which enrolled 478 patients; 1 study evaluated ipragliflozin treatment, with a cohort of 68 patients; and 4 studies analyzed the effects of sotagliflozin, which involved a total of 5,537 patients. Notably, except for empagliflozin and dapagliflozin, no studies directly compared the remaining four types of SGLT2i.

**Table 1:** Characteristics of included studies.

Author, year	Design	Country	Age, year	Sample size (*n*)	EF (%)	Type of SGLT2i	Control	Follow-up (months)	Oral dose (mg)	Outcome
Savarese, 2021	RCT	Global	65.45 ± 9.41	208	≥50	Empagliflozin	Placebo	37.2	10/25	ABCD
Ueda, 2021	RCT	Canada	75.70 ± 6.50	82	≥50	Canagliflozin	Placebo	5.6	100	BE
Akasaka, 2022	RCT	Japan	71.14 ± 8.21	68	≥50	Ipragliflozin	Placebo	2.8	NR	JKLMN
Bhatt, 2021	RCT	Global	69.00 ± 1.83	1,667	≥50	Sotagliflozin	Placebo	16	200/400	ABCDE
Pu XP, 2022	RCT	China	74.92 ± 8.57	123	≥50	Empagliflozin	Placebo	12	10	BFGIJK
Li L, 2021	RCT	China	61.40 ± 9.78	60	≥50	Dapagliflozin	Placebo	6	10	EFIJ
Liu SS, 2022	RCT	China	72.0 ± 6.0	100	≥50	Dapagliflozin	Placebo	12	10	GN
Luo P, 2022	RCT	China	65.47 ± 7.15	64	≥50	Dapagliflozin	Placebo	6	10	EFGN
Sun H, 2021	RCT	China	71.0 ± 7.38	46	≥50	Dapagliflozin	Placebo	6	10	GJ
Xu X, 2021	RCT	China	67.01 ± 6.59	100	≥50	Dapagliflozin	Placebo	6	10	EN
Yang F, 2022	RCT	China	18–80	96	≥50	Dapagliflozin	Placebo	6	10	GHN
Solomon, 2022	RCT	Global	71.6 ± 9.5	4,147	≥50	Dapagliflozin	Placebo	8	10	ABCD
Anker, 2021	RCT	Global	71.8 ± 9.3	4,013	≥50	Empagliflozin	Placebo	26.2	10	ABCDE
Oldgren, 2021	RCT	Italy	64.4	49	≥50	Dapagliflozin	Placebo	1.5	10	GIKCMNO
Tanaka, 2020	RCT	Japan	66.42 ± 10.18	165	≥50	Canagliflozin	Placebo	6	10	GJN
Zeng H, 2023	RCT	China	52.10 ± 6.06	100	≥50	Dapagliflozin	Placebo	6	10	GLMN
Duan HQ, 2023	RCT	China	52.92 ± 10.32	90	≥50	Dapagliflozin	Placebo	5.6	10	ABCDGIJK
Liang ML, 2023	RCT	China	70.26 ± 0.53	80	≥50	Dapagliflozin	Placebo	3	10	IK
Liu SS, 2023	RCT	China	64.03 ± 5.25	87	≥50	Dapagliflozin	Placebo	6	10	GKLM
Lv LX, 2023	RCT	China	75.12 ± 5.45	115	≥50	Dapagliflozin	Placebo	6	10	GIKN
Wang JM, 2023	RCT	China	18–75	74	≥50	Dapagliflozin	Placebo	12	10	LM
Zhang N, 2024	RCT	China	72.30 ± 3.29	200	≥50	Dapagliflozin	Placebo	6	10	CEFGN
Savarese, 2021	RCT	Global	64.03 ± 8.59	419	<50	Empagliflozin	Placebo	37.2	10	ABCD
Anker, 2021	RCT	Global	69.99 ± 9.84	1983	40–50	Empagliflozin	Placebo	31.2	10	ABCD
Bhatt, 2021	RCT	Global	69.00 ± 1.83	2,108	≤40	Sotagliflozin	Placebo	16	200/400	ABCDE
Bhatt, 2021	RCT	Global	68.15 ± 2.25	966	<50	Sotagliflozin	Placebo	9	200/400	A
Nassif, 2019	RCT	USA	60.61 ± 11.98	263	≤40	Dapagliflozin	Placebo	2.8	10	ACFGH
McMurray, 2019	RCT	Global	64.34 ± 11.01	4,744	≤40	Dapagliflozin	Placebo	2	10	ABCD
Jensen, 2020	RCT	Denmark	64.00 ± 11.00	190	≤40	Empagliflozin	Placebo	2.8	10	GH
Packer, 2020	RCT	Global	66.78 ± 11.00	3,730	≤40	Empagliflozin	Placebo	16	10	ABCD
Lee, 2021	RCT	British	68.7 ± 11.1	105	≤40	Empagliflozin	Placebo	8.4	10	FGHIKLMNO
Santos, 2021	RCT	USA	62 ± 12.1	84	≤40	Empagliflozin	Placebo	6	10	CDFHILMNO
Omar, 2021	RCT	Denmark	64 ± 11	190	≤40	Empagliflozin	Placebo	2.8	10	IKLMNO
Cosentino, 2020	RCT	USA	64.33 ± 7.69	478	≤45	Ertugliflozin	Placebo	42	15	B
Abraham, 2021	RCT	Global	69.5 ± 2.41	312	≤40	Empagliflozin	Placebo	2.8	10	FH
Cao HQ, 2022	RCT	China	61.26 ± 2.66	48	≤40	Dapagliflozin	Placebo	6	10	FG
Cai RY, 2020	RCT	China	66.31 ± 6.52	80	≤40	Dapagliflozin	Placebo	6	10	GKN
Dai RX, 2022	RCT	China	66.5 ± 6.89	50	40–50	Dapagliflozin	Placebo	6	10	FGN
Deng YF, 2022	RCT	China	84.68 ± 3.67	70	≤40	Dapagliflozin	Placebo	6	10	GN
Fan H, 2022	RCT	China	68.43 ± 12.77	80	≤40	Dapagliflozin	Placebo	3	10	FGN
Ni RZ, 2023	RCT	China	71.8 ± 3.39	200	≤40	Dapagliflozin	Placebo	3	10	FGN
He GZ, 2022	RCT	China	65.08 ± 5.25	100	≤40	Dapagliflozin	Placebo	6	10	GN
Su Y, 2022	RCT	China	51.69 ± 4.05	104	≤40	Dapagliflozin	Placebo	3	10	FGN
Jia PC, 2021	RCT	China	71.32 ± 3.32	50	≤40	Dapagliflozin	Placebo	3	10	ABEGJN
Xu LH, 2023	RCT	China	63.02 ± 9.71	84	≤40	Dapagliflozin	Placebo	9	10	BN
Li XF, 2020	RCT	China	72.81 ± 8.36	102	≤40	Dapagliflozin	Placebo	2.8	10	FGN
Liu YL, 2022	RCT	China	69.33 ± 5.39	106	≤40	Dapagliflozin	Placebo	3	10	B
Zhang LN, 2023	RCT	China	65.82 ± 6.60	70	≤40	Dapagliflozin	Placebo	2.8	10	FG
Wang FB, 2023	RCT	China	61.49 ± 6.68	103	40–50	Dapagliflozin	Placebo	6	10	FGN
Yang P, 2021	RCT	China	63.10 ± 7.04	104	≤40	Dapagliflozin	Placebo	6	10	FGN
Wu WJ, 2021	RCT	China	69.00 ± 7.25	112	40–50	empagliflozin	Placebo	6	10	AEFGH
Zhang ZR, 2022	RCT	China	55.60 ± 5.21	100	40–50	Dapagliflozin	Placebo	6	10	FKLMN
Zheng HS, 2021	RCT	China	61.92 ± 11.56	147	≤40	Dapagliflozin	Placebo	12	10	EFGN
Ferreira, 2021	RCT	Global	68.20 ± 9.66	3,726	≤40	Empagliflozin	Placebo	12	10	ABCD
Jensen, 2021	RCT	Denmark	67.5 ± 10	120	≤40	Empagliflozin	Placebo	2.8	10	EO
Packer, 2021	RCT	Global	66.7 ± 10.9	3,726	≤40	Empagliflozin	Placebo	12.1	10	ABC
Palau, 2022	RCT	Spain	67.1 ± 10.7	90	≤40	Dapagliflozin	Placebo	3	10	F
Solomon, 202	RCT	Global	71.7 ± 9.5	2,116	<50	Dapagliflozin	Placebo	8	10	A
Anker, 2021	RCT	Global	71.7 ± 9.21	1,983	<50	Empagliflozin	Placebo	26.2	10	A
Gao ML, 2022	RCT	China	50.55 ± 4.50	123	<50	Dapagliflozin	Placebo	6	10	FGN
Tanaka, 2020	RCT	Japan	66.42 ± 10.18	68	<50	Canagliflozin	Placebo	6	100	GJN
Carbone, 2020	RCT	USA	58.15 ± 7.54	36	<50	Canagliflozin	Placebo	3	100	JLMN
Wei YJ, 2020	RCT	China	60.5 ± 13.53	102	<50	Canagliflozin	Placebo	2	10	FGN
Chen A, 2023	RCT	China	64.81 ± 10.72	80	≤40	Dapagliflozin	Placebo	12	10	ABCDG
Du BY, 2023	RCT	China	62.59 ± 5.67	75	≤40	Dapagliflozin	Placebo	2.8	10	CGN
Gong ZY, 2023	RCT	China	65.44 ± 4.68	100	≤40	Dapagliflozin	Placebo	6	10	BFGN
He P, 2023	RCT	China	68.75 ± 3.53	76	<50	Dapagliflozin	Placebo	6	10	GN
lv G, 2023	RCT	China	58.95 ± 6.26	160	≤40	Dapagliflozin	Placebo	12	10	GN
Pan LH, 2023	RCT	China	69.07 ± 9.35	120	≤40	Dapagliflozin	Placebo	2.8	10	FHN
Peng XX, 2023	RCT	China	59.97 ± 2.10	68	40–50	Dapagliflozin	Placebo	6	10	IJKL
Wu F, 2023	RCT	China	61.01 ± 4.03	159	≤40	Dapagliflozin	Placebo	3	10	GN
Wu JF, 2023	RCT	China	69.28 ± 7.19	60	≤40	Dapagliflozin	Placebo	3	10	FGHLM
Wu N, 2023	RCT	China	64.8 ± 4.19	60	≤40	Dapagliflozin	Placebo	6	10	FGN
Yang L, 2023	RCT	China	67.32 ± 5.54	80	≤40	Dapagliflozin	Placebo	3	10	BCDGN
Zhao CC, 2023	RCT	China	55.89 ± 8.26	60	≤40	Dapagliflozin	Placebo	3	10	GN
Chen Y, 2024	RCT	China	65.14 ± 5.41	80	≤45	Dapagliflozin	Placebo	3	10	FGN
Gong QP, 2024	RCT	China	65.50 ± 5.48	120	≤40	Dapagliflozin	Placebo	12	10	EGN
Lu Q, 2024	RCT	China	67.43 ± 1.45	128	≤40	Dapagliflozin	Placebo	12	10	ACDEGN
Zhang LF, 20	RCT	China	72.95 ± 8.95	60	≤40	Dapagliflozin	Placebo	5	10	FGN
Bertram, 2023	RCT	USA	69.12 ± 2.00	596	<50	Sotagliflozin	Placebo	3	200	ABCDE
McMurray, 2024	RCT	British	69.00 ± 2.33	313	≤40	Dapagliflozin	Placebo	3.7	10	FH
Fu QY, 2023	RCT	China	70.55 ± 6.44	60	≤40	Dapagliflozin	Placebo	12	10	LMN

### Bias risk evaluation

**Figure 2:**
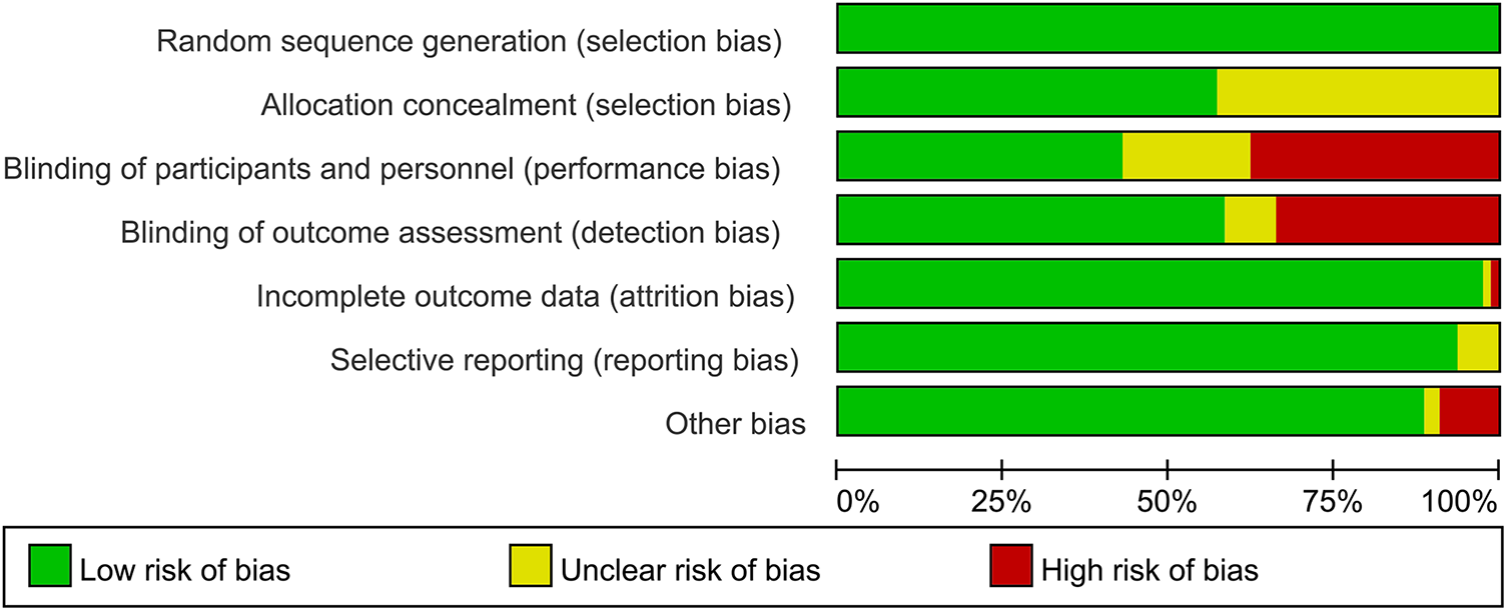
Risk of bias summary of all eligible RCTs evaluating the effect of SGLT2i in HFrEF or HFpEF.

The results of the bias risk evaluation are presented in Figure 2 and Supplementary Figure S1. For random sequence generation, 77 studies employing a random number table or a random Excel table were identified as low risk; 33 studies provided detailed descriptions of their allocation concealment procedures; however, the remaining studies lacked such descriptions. Regarding implementation bias, 29 studies were defined as high risk, 15 studies did not provide sufficient details, and the rest were classified as low risk. For the assessment of outcome data, one study was identified as high risk due to insufficient details, one was poorly described, and the others were considered low risk. All studies reported complete data for outcomes. Outcome selection bias indicated that 72 studies were classified as low risk, while 5 required further clarification. The results of other preferences showed that seven studies were defined as high risk, two were poorly described, and the remaining studies were assessed as low risk.

### Meta-analysis results

#### A composite of hospitalizations for HF and CV death

**Figure 3:**
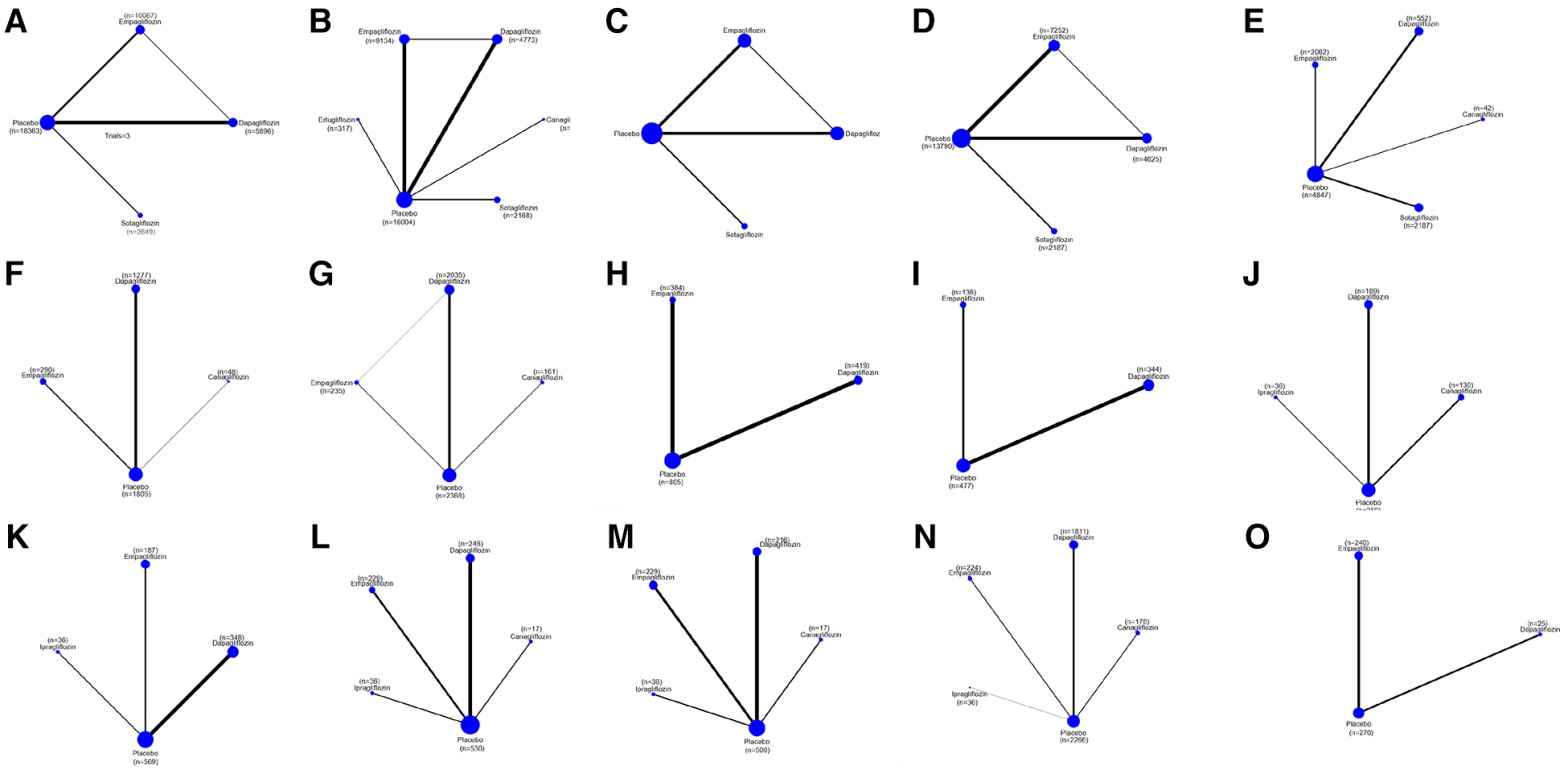
A network plot of each comparison in all eligible trials in HFrEF or HFpEF. (**A**) The network plot of each comparison in terms of a composite of hospitalization for HF and CV death. (**B**) The network plot of each comparison in terms of hospitalization for HF. (**C**) The network plot of each comparison in terms of CV death. (**D**) The network plot of each comparison in terms of all-cause death. (**E**) The network plot of each comparison in terms of a composite of urinary and reproductive infections. (**F**) The network plot of each comparison in terms of 6 min walk distance. (**G**) The network plot of each comparison in terms of NT-proBNP. (**H**) The network plot of each comparison in terms of KCCQ. (**I**) The network plot of each comparison in terms of LAVi. (**J**) The network plot of each comparison in terms of E/e’. (**K**) The network plot of each comparison in terms of LVMi. (**L**) The network plot of each comparison in terms of LVEDV. (**M**) The network plot of each comparison in terms of LVESV. (**N**) The network plot of each comparison in terms of LVEF. (**O**) The network plot of each comparison in terms of HCT.

As shown in Table 2, sotagliflozin [RR = 0.69, 95% CI (0.64–0.75)], empagliflozin [RR = 0.79, CI (0.75–0.84)], and dapagliflozin [RR = 0.80, CI:(0.75–0.87)] exhibited significant efficacy in reducing a composite outcome of hospitalization for HF and CV death when compared to placebo. Sotagliflozin demonstrated superiority over empagliflozin [RR = 0.88, CI (0.79–0.97)] and dapagliflozin [RR = 0.86, CI (0.77–0.96)]. The network plot is shown in Figure 3A. The ranking based on SUCRA values is as follows: sotagliflozin (99%), empagliflozin (55%), dapagliflozin (47%), and placebo (0%), as shown in Table 3.

**Table 2:** Comparison of the efficacy and safety of different SGLT2i in treating HF [RR and mean difference (95% CI)].

A composite of hospitalization for HF and CV death					
Sotagliflozin					
**0.88 (0.79, 0.97)**	Empagliflozin				
**0.86 (0.77, 0.96)**	0.98 (0.89, 1.08)	Dapagliflozin			
**0.69 (0.64, 0.75)**	**0.79 (0.75, 0.84)**	**0.80 (0.75, 0.87)**	Placebo		
Hospitalization for HF					
Dapagliflozin					
0.94 (0.52, 1.69)	Sotagliflozin				
0.90 (0.37, 2.20)	0.96 (0.37, 2.45)	Ertugliflozin			
0.60 (0.04, 10.12)	0.64 (0.04, 10.94)	0.67 (0.04, 12.38)	Canagliflozin		
0.75 (0.49, 1.16)	0.80 (0.47, 1.37)	0.84 (0.35, 1.99)	1.26 (0.08, 21.19)	Empagliflozin	
**0.57 (0.39, 0.82)**	**0.61 (0.38, 0.96)**	0.63 (0.28, 1.44)	0.95 (0.06, 15.75)	**0.75 (0.58, 0.99)**	Placebo
CV death					
Dapagliflozin					
0.97 (0.66, 1.44)	Sotagliflozin				
0.93 (0.73, 1.19)	0.96 (0.66, 1.38)	Empagliflozin			
0.84 (0.68, 1.03)	0.86 (0.61, 1.21)	0.90 (0.78, 1.03)	Placebo		
All-cause death					
Empagliflozin					
1.00 (0.81, 1.24)	Dapagliflozin				
0.89 (0.60, 1.31)	0.89 (0.59, 1.33)	Sotagliflozin			
0.89 (0.79, 1.01)	0.89 (0.75, 1.06)	1.01 (0.70, 1.45)	Placebo		
A composite of urinary and reproductive infections					
Canagliflozin					
0.12 (0.00, 3.09)	Sotagliflozin				
0.11 (0.00, 2.48)	0.89 (0.39, 2.01)	Placebo			
0.08 (0.00, 2.16)	0.65 (0.17, 2.48)	0.73 (0.26, 2.11)	Empagliflozin		
0.05 (0.00, 1.30)	0.43 (0.15, 1.26)	**0.49 (0.24, 0.97)**	0.66 (0.19, 2.34)	Dapagliflozin	
6MWT					
Canagliflozin					
21.07 (−60.18, 102.31)	Dapagliflozin				
31.27 (−58.25, 120.78)	10.20 (−32.77, 53.17)	Empagliflozin			
71.62 (−8.36, 151.60)	**50.55 (35.27, 65.84)**	**40.35 (0.19, 80.52)**	Placebo		
NT-proBNP					
Dapagliflozin					
171.98 (−320.51, 664.46)	Empagliflozin				
−205.67 (−602.26, 190.91)	−377.65 (−992.37, 237.07)	Canagliflozin			
**−365.33 (−504.83, −225.83)**	**−537.31 (−1,021.40, −53.22)**	−159.66 (−543.13, 223.81)	Placebo		
KCCQ					
Dapagliflozin					
1.10 (−2.63, 4.84)	Empagliflozin				
**4.72 (2.52, 6.91)**	**3.61 (0.61, 6.62)**	Placebo			
LAVi					
Dapagliflozin					
−2.74 (−6.37, 0.90)	Empagliflozin				
**−2.67 (−3.44, −1.91)**	0.06 (−3.50, 3.63)	Placebo			
E/e’					
Dapagliflozin					
−0.73 (−3.52, 2.07)	Placebo				
−1.13 (−8.26, 6.00)	−0.40 (−6.96, 6.16)	Ipragliflozin			
−3.80 (−8.41, 0.81)	−3.07 (−6.96, 0.81)	−2.67 (−10.30, 4.95)	Canagliflozin		
LVMi					
Ipragliflozin					
−12.16 (−39.49, 15.17)	Empagliflozin				
−14.58 (−41.45, 12.28)	−2.42 (−8.51, 3.66)	Dapagliflozin			
−19.10 (−45.86, 7.66)	**−6.94 (−12.52, −1.36)**	**−4.52 (−6.96, −2.08)**	Placebo		
LVEDV					
Canagliflozin					
−3.81 (−25.25, 17.63)	Dapagliflozin				
−8.17 (−32.21, 15.87)	−4.36 (−21.51, 12.79)	Empagliflozin			
−9.71 (−44.29, 24.87)	−5.90 (−35.87, 24.08)	−1.54 (−33.58, 30.50)	Ipragliflozin		
−16.41 (−35.96, 3.14)	**−12.60 (−21.83, −3.37)**	−8.24 (−22.85, 6.37)	−6.70 (−35.22, 21.82)	Placebo	
LVESV					
Canagliflozin					
−4.34 (−19.88, 11.21)	Dapagliflozin				
−7.12 (−29.68, 15.45)	−2.78 (−21.72, 16.16)	Ipragliflozin			
−8.30 (−25.59, 8.98)	−3.97 (−16.21, 8.28)	−1.19 (−21.80, 19.43)	Empagliflozin		
−13.32 (−27.26, 0.63)	**−8.98 (−15.64, −2.33)**	−6.20 (−23.94, 11.54)	−5.02 (−15.53, 5.49)	Placebo	
LVEF					
Dapagliflozin					
0.70 (−3.41, 4.82)	Empagliflozin				
1.67 (−2.90, 6.23)	0.96 (−4.91, 6.84)	Canagliflozin			
2.95 (−5.46, 11.35)	2.24 (−6.95, 11.44)	1.28 (−8.13, 10.69)	Ipragliflozin		
**5.05 (3.81, 6.29)**	**4.34 (0.42, 8.27)**	3.38 (−1.02, 7.78)	2.10 (−6.22, 10.42)	Placebo	
HCT					
Placebo					
−0.01 (−0.02, 0.00)	Dapagliflozin				
**−0.03 (−0.04, −0.02)**	−0.02 (−0.03, −0.00)	Empagliflozin			

In bold: values of statistical significance (*P *< 0.05).

**Table 3:** Ranking probability of the efficacy of drug in patients with HF.

Ranking	A composite of hospitalization for HF and CV death	Hospitalization for HF	CV death	All-cause death	A composite of urinary and reproductive infections	6MWT	NT-ProBNP	KCCQ	LAVi	E/e’	LVMi	LVEDV	LVESV	LVEF	HCT
1	Sotagliflozin (99%)	Dapagliflozin (74%)	Sotagliflozin (81%)	Dapagliflozin (74%)	Canagliflozin (92%)	Canagliflozin (80%)	Empagliflozin (87%)	Dapagliflozin (86%)	Dapagliflozin (96%)	Dapagliflozin (75%)	Ipragliflozin (85%)	Canagliflozin (76%)	Canagliflozin (80%)	Dapagliflozin (78%)	Placebo (95%)
2	Empagliflozin (55%)	Sotagliflozin (67%)	Dapagliflozin (77%)	Sotagliflozin (61%)	Sotagliflozin (60%)	Dapagliflozin (65%)	Dapagliflozin (70%)	Empagliflozin (63%)	Empagliflozin (27%)	Placebo (60%)	Empagliflozin (66%)	Dapagliflozin (67%)	Dapagliflozin (65%)	Empagliflozin (66%)	Dapagliflozin (53%)
3	Dapagliflozin (45%)	Ertugliflozin (59%)	Empagliflozin (35%)	Empagliflozin (55%)	Placebo (54%)	Empagliflozin (51%)	Canagliflozin (34%)	Placebo (1%)	Placebo (25%)	Ipragliflozin (52%)	Dapagliflozin (45%)	Empagliflozin (49%)	Ipragliflozin (43%)	Canagliflozin (53%)	Empagliflozin (1%)
4	Placebo (0%)	Empagliflozin (43%)	Placebo (6%)	Placebo (10%)	Empagliflozin (34%)	Placebo (2%)	Placebo (8%)			Canagliflozin (11%)	Placebo (3%)	Ipragliflozin (44%)	Empagliflozin (48%)	Ipragliflozin (41%)	
5		Canagliflozin (41%)			Dapagliflozin (10%)							Placebo (12%)	Placebo (11%)	Placebo (10%)	
6		Placebo (13%)													

### Hospitalization for HF

As shown in Table 2, empagliflozin [RR = 0.75, CI (0.58–0.99)], sotagliflozin (RR = 0.61, CI 0.38–0.96), and dapagliflozin [RR = 0.57, CI (0.39–0.82)] significantly reduced hospitalization for HF compared to placebo. However, no significant differences were observed between ertugliflozin and canagliflozin or among the different SGLT2i treatments. The network plot is shown in Figure 3B. The ranking based on SUCRA values is as follows: dapagliflozin (74%), sotagliflozin (67%), erugliflozin (59%), empagliflozin (43%), canagliflozin (41%), and placebo (13%).

### CV death and all-cause death

As shown in Table 2, empagliflozin, dapagliflozin, and sotagliflozin showed no difference in reducing all-cause mortality and cardiovascular mortality compared to placebo. The network plot is shown in Figure 3D, while the ranking based on SUCRA values is presented in Table 3.

### A composite of urinary and reproductive infections

As shown in Table 2, dapagliflozin [RR = 0.49, CI (0.24–0.97)] significantly reduced urinary and reproductive system infections compared to placebo. However, canagliflozin, sotagliflozin, and empagliflozin did not exhibit a significant difference in reducing these infections. There was no difference observed among the different SGLT2i treatments. These findings are visually represented in the network plot shown in Figure 3E. The ranking based on SUCRA value is as follows: canagliflozin (92%), sotagliflozin (60%), placebo (54%), empagliflozin (34%), and dapagliflozin (10%).

### 6MWT

Table 2 shows that dapagliflozin [RR = 50.55, CI (35.27–65.84)] and empagliflozin [RR = 40.35, CI (0.19–80.52)] significantly improved walking distance compared to placebo, whereas canagliflozin [RR = 71.62, CI (−8.36–151.60)] showed no difference. There was no difference observed among the different SGLT2i treatments. These comparisons are graphically illustrated in the network plot shown in Figure 3F. The ranking based on SUCRA values is as follows: canagliflozin (80%), dapagliflozin (65%), empagliflozin (51%), and placebo (2%).

### NT-proBNP

As presented in Table 2, empagliflozin [MD = −537.81, CI (−1,021.40 to −53.22)] and dapagliflozin [MD = −365.33, CI (−504.83 to −225.83)] showed significant differences in improving NT-proBNP in patients with HF compared to placebo. However, canagliflozin [MD = −159.66, CI (−543.13–223.81)] showed no significant impact on proBNP levels. There was no difference observed among the different SGLT2i treatments. These results are illustrated in the network plot shown in Figure 3G. The ranking based on SUCRA values is as follows: empagliflozin (87%), dapagliflozin (70%), canagliflozin (34%), and placebo (8%).

### KCCQ

As detailed in Table 2, compared to placebo, there was no significant difference in the improvement of KCCQ scores in patients with HF between dapagliflozin [MD = 4.72, CI (2.52–6.91)] and empagliflozin [MD = 3. 6, CI (0.61–6.62)]. No significant differences were observed among the different SGLT2i treatments. These findings are visually represented in the network plot shown in Figure 3H. The ranking based on SUCRA values is as follows: dapagliflozin 86%), empagliflozin (63%), and placebo (1%).

### LAVi

As shown in Table 2, compared with placebo, dapagliflozin [MD = −2.67, CI (−3.44 to −1.91)] showed significant statistical differences in improving LAVi, while empagliflozin [MD = 0.06, CI (−3.05–3.63)] showed no significant change. These findings are depicted in the network plot shown in Figure 3I. The ranking based on SUCRA values is as follows: dapagliflozin (96%), empagliflozin (27%), and placebo (25%).

### E/e'

As shown in Table 2, compared with placebo, dapagliflozin [MD = −0.73, CI (−3.52–2.07)], ipragliflozin [MD = −0.40, CI (−6.96–6.16)], and canagliflozin [MD = −3.07, CI (−6.96–0.81)] showed no difference in improving E/e', and there were no significant variations observed among the different SGLT2i treatments. The network plot is presented in Figure 3J. The ranking based on SUCRA values is as follows: dapagliflozin (75%), placebo (60%), ipragliflozin (52%), and canagliflozin (11%).

### LVMi

The results presented in Table 2 indicated that empagliflozin [MD = −6.94, CI (−12.52 to −1.36)] and dapagliflozin [MD = −4.52, CI (−6.96 to −2.08)] significantly reduced LVMi compared to placebo. There was no significant difference observed among the different SGLT2i treatments. The network plot is shown in Figure 3K. The ranking based on SUCRA values is as follows: ipragliflozin (85%), empagliflozin (66%), dapagliflozin (45%), and placebo (3%).

### LVEDV

Table 2 demonstrates that dapagliflozin [MD = −12.60, CI (−21.83 to −3.37)] reduced LVEDV in patients with HF compared to placebo, while ipragliflozin, empagliflozin, and canagliflozin did not show a significant impact in this aspect. No differences were observed among the different SGLT2i treatments. The network plot illustrating these findings is presented in Figure 3L. The ranking based on SUCRA values is as follows: canagliflozin (76%), empagliflozin (67%), ipragliflozin (49%), dapagliflozin (44%), and placebo (12%).

### LVESV

Table 2 demonstrates that dapagliflozin [MD = −8.98, CI (−15.64 to −2.33)] reduced LVESV in patients with HF compared to placebo. In contrast, ipragliflozin, canagliflozin, and empagliflozin did not exhibit statistically significant differences in this aspect, indicating no significant variance among the different SGLT2i treatments. The network plot illustrating these relationships is presented in Figure 3M. The ranking based on SUCRA values is as follows: canagliflozin (80%), dapagliflozin (65%), ipragliflozin (43%), empagliflozin (48%), and placebo (11%).

### LVEF

As indicated in Table 2, dapagliflozin [MD = 5.05, CI (3.81–6.29)] significantly increased LVEF in patients with HF compared to placebo, whereas canagliflozin, empagliflozin, and ipragliflozin showed no significant differences. No differences were observed among the different SGLT2i treatments. These findings are visually represented in the network plot shown in Figure 3N. The ranking based on SUCRA values is as follows: dapagliflozin (78%), empagliflozin (66%), canagliflozin (53%), ipragliflozin (41%), and placebo (10%).

### HCT

Table 2 illustrates that empagliflozin [MD = −0.03, CI (−0.04 to −0.02)] significantly increased hematocrit (HCT) in patients with HF compared to placebo. Dapagliflozin did not show any significant difference, and there was no disparity observed among the different SGLT2i treatments. The network plot illustrating these findings is presented in Figure 3O. The ranking based on SUCRA values is as follows: placebo (95%), dapagliflozin (53%), and empagliflozin (1%).

### The results of the subgroup analysis

#### The efficacy of SGLT2i in HFrEF patients

**Figure 4:**
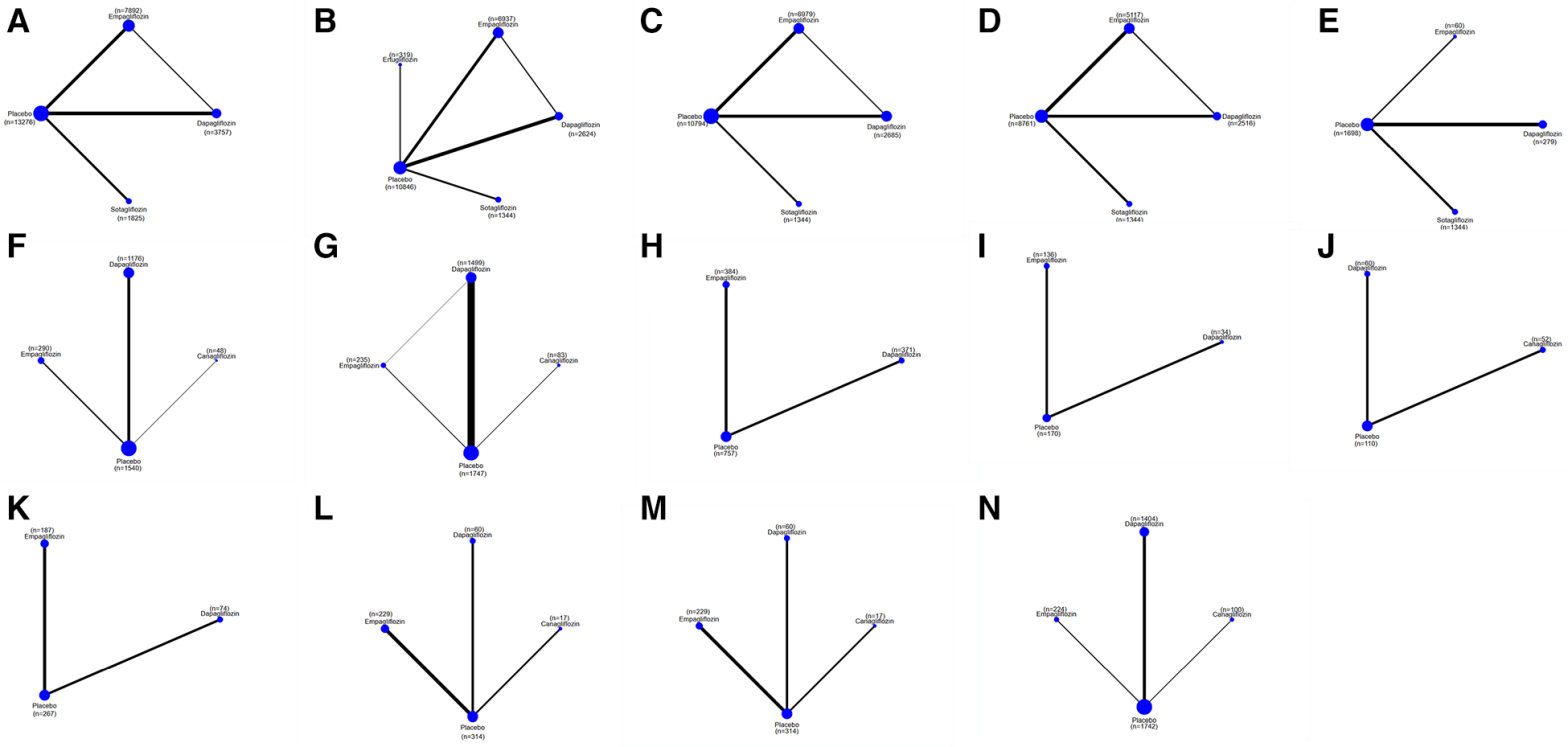
A network plot of each comparison in all eligible trials in HFrEF. (**A**) The network plot of each comparison in terms of a composite of hospitalization for HF and CV death. (**B**) The network plot of each comparison in terms of hospitalization for HF. (**C**) The network plot of each comparison in terms of CV death. (**D**) The network plot of each comparison in terms of all-cause death. (**E**) The network plot of each comparison in terms of a composite of urinary and reproductive infections. (**F**) The network plot of each comparison in terms of 6 min walk distance. (**G**) The network plot of each comparison in terms of NT-proBNP. (**H**) The network plot of each comparison in terms of KCCQ. (**I**) The network plot of each comparison in terms of LAVi. (**J**) The network plot of each comparison in terms of E/e’. (**K**) The network plot of each comparison in terms of LVMi. (**L**) The network plot of each comparison in terms of LVEDV. (**M**) The network plot of each comparison in terms of LVESV. (**N**) The network plot of each comparison in terms of LVEF.

The network plot in Figure 4 illustrates that dapagliflozin [RR = 0.44, CI (0.15–1.23)], empagliflozin [RR = 0.75, CI (0.11–5.15)], and sotagliflozin [RR = 0.42, CI (0.05–3.66)] did not significantly reduce the composite of hospitalization for HF and CV death compared to placebo, and there was no difference between the different SGLT2i, as shown in Table 4. The ranking based on SUCRA values is presented in Table 5. Compared with placebo, dapagliflozin significantly improved hospitalization for HF [RR = 0.51, CI (0.33–0.80)] and CV death [RR = 0.73, CI (0.54–0.97)], with no significant differences noted between the different SGLT2i treatments. These findings are outlined in Table 4, and the associated SUCRA rankings are presented in Table 5. Compared to placebo, dapagliflozin reduced all-cause death [RR = 0.69, CI (0.48–0.99)], and dapagliflozin [RR = 0.40, CI (0.18–0.93)] increased a composite of urinary and reproductive infections, with no differences observed between the different SGLT2i treatments. The SUCRA values for these comparisons are also presented in Table 5.

**Table 4:** Comparison of the efficacy and safety of different SGLT2i in treating HFrEF [RR and mean difference (95% CI)].

Hospitalization for HF				
Dapagliflozin				
0.85 (0.41, 1.77)	Sotagliflozin			
0.81 (0.31, 2.12)	0.95 (0.34, 2.69)	Ertugliflozin		
0.62 (0.37, 1.05)	0.73 (0.38, 1.44)	0.77 (0.31, 1.92)	Empagliflozin	
**0.51 (0.33, 0.80)**	0.60 (0.34, 1.08)	0.63 (0.27, 1.49)	0.82 (0.59, 1.14)	Placebo
A composite of urinary and reproductive infections				
Canagliflozin				
0.14 (0.00, 4.05)	Sotagliflozin			
0.11 (0.00, 2.61)	0.77 (0.26, 2.28)	Placebo		
0.08 (0.00, 2.34)	0.57 (0.12, 2.74)	0.74 (0.23, 2.31)	Empagliflozin	
0.04 (0.00, 1.18)	0.31 (0.08, 1.23)	**0.40 (0.18, 0.93)**	0.55 (0.13, 2.27)	Dapagliflozin
A composite of hospitalization for HF and CV death				
Sotagliflozin				
0.57 (0.22, 1.48)	Placebo			
0.42 (0.05, 3.66)	0.75 (0.11, 5.15)	Empagliflozin		
0.25 (0.06, 1.01)	0.44 (0.15, 1.23)	0.58 (0.07, 5.17)	Dapagliflozin	
CV death				
Dapagliflozin				
0.94 (0.53, 1.67)	Sotagliflozin			
0.77 (0.55, 1.07)	0.81 (0.48, 1.37)	Empagliflozin		
0.73 (0.54, 0.97)	0.77 (0.47, 1.27)	0.95 (0.81, 1.11)	Placebo	
All-cause death				
Dapagliflozin				
0.89 (0.51, 1.57)	Sotagliflozin			
0.78 (0.51, 1.19)	0.87 (0.52, 1.45)	Empagliflozin		
**0.69 (0.48, 0.99)**	0.77 (0.48, 1.23)	0.88 (0.73, 1.08)	Placebo	
6MWT				
Canagliflozin				
27.63 (−46.83, 102.09)	Dapagliflozin			
29.24 (−52.88, 111.36)	1.61 (−38.08, 41.30)	Empagliflozin		
69.24 (−4.02, 142.51)	**41.61 (27.23, 56.00)**	**40.00 (2.99, 77.01)**	Placebo	
NT-proBNP				
Empagliflozin				
−144.64 (−675.00, 385.71)	Dapagliflozin			
−376.49 (−1,113.11, 360.12)	−231.85 (−776.49, 312.79)	Canagliflozin		
**−547.13 (−1,065.35, −28.91)**	**−402.49 (−575.29, −229.68)**	−170.64 (−699.36, 358.08)	Placebo	
LVEF				
Dapagliflozin				
1.79 (−2.35, 5.92)	Empagliflozin			
1.35 (−4.04, 6.74)	−0.44 (−6.92, 6.04)	Canagliflozin		
**5.64 (4.27, 7.01)**	3.85 (−0.05, 7.76)	4.29 (−0.93, 9.51)	Placebo	
KCQQ				
Dapagliflozin				
1.27 (−3.28, 5.82)	Empagliflozin			
**4.86 (1.90, 7.83)**	**3.60 (0.15, 7.04)**	Placebo		
E/e’				
Dapagliflozin				
−0.28 (−7.87, 7.31)	Placebo			
−4.60 (−13.93, 4.74)	−4.32 (−11.37, 2.73)	Canagliflozin		
LVMi				
Empagliflozin				
−3.09 (−7.42, 1.23)	Dapagliflozin			
**−6.89 (−11.18, −2.59)**	**−3.79 (−4.32, −3.27)**	Placebo		
LVEDV				
Dapagliflozin				
−1.65 (−29.15, 25.86)	Canagliflozin			
−10.92 (−34.09, 12.25)	−9.27 (−33.65, 15.10)	Empagliflozin		
**−19.14 (−38.14, −0.13)**	−17.49 (−37.30, 2.32)	−8.22 (−22.73, 6.29)	Placebo	
LVESV				
Dapagliflozin				
−0.94 (−20.48, 18.60)	Canagliflozin			
−9.95 (−25.37, 5.47)	−9.01 (−26.23, 8.20)	Empagliflozin		
**−14.78 (−27.79, −1.78)**	**−13.85 (−27.33, −0.36)**	−4.84 (−15.19, 5.52)	Placebo	

In bold: values of statistical significance (*P *< 0.05).

**Table 5:** Ranking probability of the efficacy of drug in patients with HFrEF.

Ranking	A composite of hospitalization for HF and CV death	Hospitalization for HF	CV death	All-cause death	A composite of urinary and reproductive infections	6MWT	NT-proBNP	LAVi	KCCQ	E/e’	LVMi	LVEDV	LVESV	LVEF
1	Sotagliflozin (97%)	Dapagliflozin (82%)	Dapagliflozin (75%)	Dapagliflozin (83%)	Sotagliflozin (87%)	Canagliflozin (82%)	Empagliflozin (84%)	Empagliflozin (77%)	Dapagliflozin (85%)	Dapagliflozin (68%)	Empagliflozin (96%)	Dapagliflozin (78%)	Dapagliflozin (80%)	Dapagliflozin (82%)
2	Empagliflozin (51%)	Sotagliflozin (65%)	Empagliflozin (50%)	Empagliflozin (63%)	Placebo (57%)	Dapagliflozin (59%)	Dapagliflozin (69%)	Dapagliflozin (71%)	Empagliflozin (63%)	Placebo (67%)	Dapagliflozin (53%)	Canagliflozin (72%)	Canagliflozin (76%)	Canagliflozin (61%)
3	Dapagliflozin (47%)	Ertugliflozin (59%)	Sotagliflozin (49%)	Sotagliflozin (44%)	Empagliflozin (42%)	Empagliflozin (57%)	Canagliflozin (36%)	Canagliflozin (41%)	Placebo (2%)	Canagliflozin (13%)	Placebo (1%)	Empagliflozin (42%)	Empagliflozin (36%)	Empagliflozin (54%)
4	Placebo (0%)	Empagliflozin (34%)	Placebo (24%)	Placebo (9%)	Dapagliflozin (13%)	Placebo (2%)	Placebo (9%)	Placebo (9%)				Placebo (7%)	Placebo (7%)	Placebo (3%)
5		Placebo (8%)												

Compared to placebo, dapagliflozin improved the 6MWT of HFrEF patients [MD = 41.61, CI (27.23–56.00)] and proBNP levels [MD = −402.49, CI (−575.29 to −299.68)], and empagliflozin improved the 6MWT of HF patients [MD = 40.00, CI (2.99–77.01)] and proBNP levels [MD = −547.13, CI (−1,065.35 to −28.91)]. No significant differences were observed between different SGLT2i. The ranking based on SUCRA values is shown in Table 5. Compared to placebo, SGLT2i did not improve E/e' in cardiac structure. Compared to placebo, dapagliflozin significantly reduced LVMi [MD = −3.79, CI (−4.33 to −3.27)] and increased LVEF [MD = 5.64, CI (4.27 to 7.01)] LVEDV [MD = −19.14, CI (−38.14 to −0.13)], LVESV [MD = −14.78, CI (−27.79 to −1.78)], and KCCQ score [MD = 4.86, CI (1.90–7.83)]. Compared to placebo, canagliflozin reduced LVESV [MD = −13.85, CI (−27.33 to −0.36] in patients with HFrEF. Compared to placebo, empagliflozin reduced LVMi [MD = −6.89, CI (−11.18 to −2.59)] and KCCQ score [MD = 3.60, CI (0.15–7.04)]. The ranking based on SUCRA values is shown in Table 5.

#### The efficacy of SGLT2i in HFpEF patients

**Figure 5:**
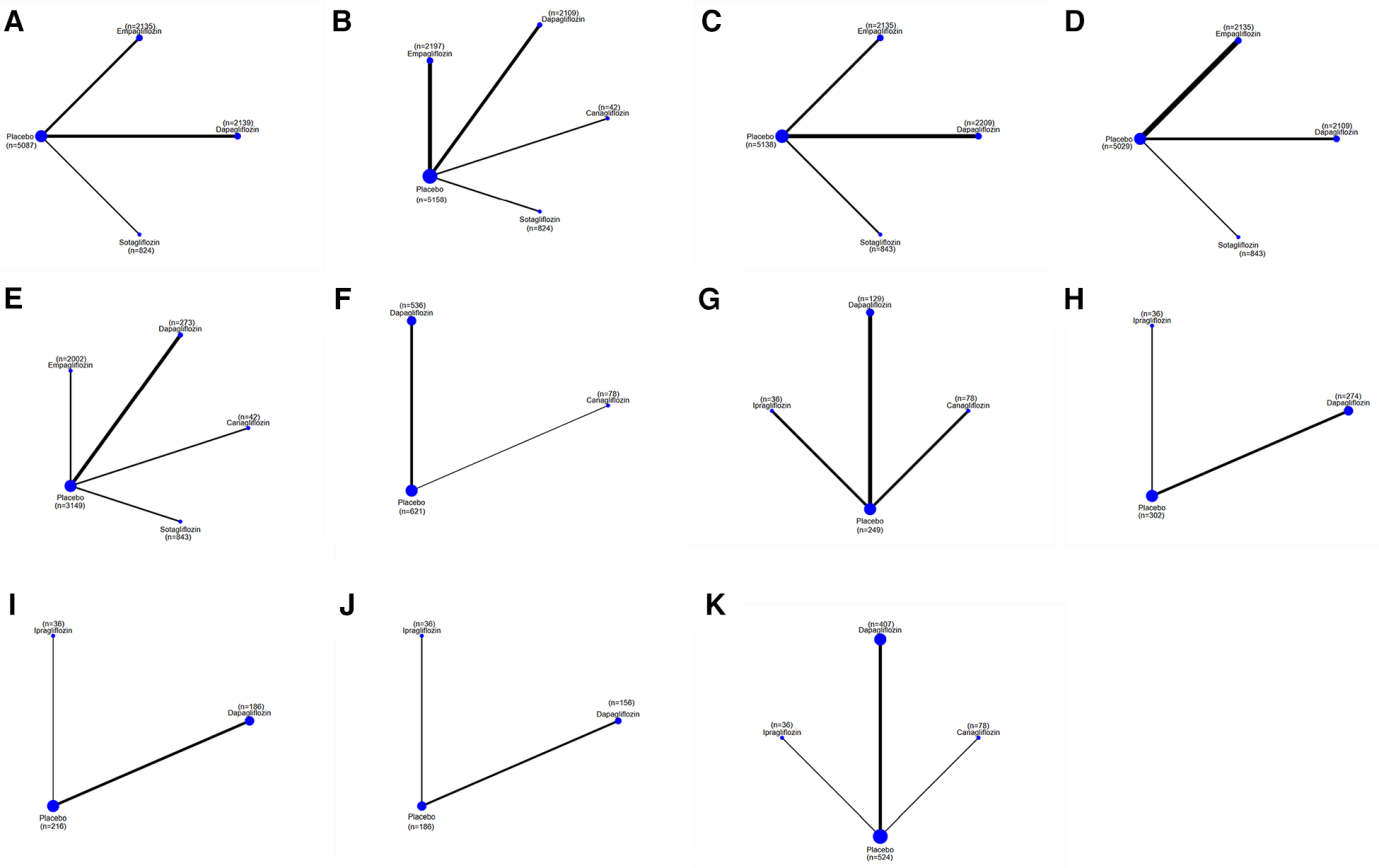
A network plot of each comparison in all eligible trials in HFpEF. (**A**) The network plot of each comparison in terms of a composite of hospitalization for HF and CV death. (**B**) The network plot of each comparison in terms of hospitalization for HF. (**C**) The network plot of each comparison in terms of CV death. (**D**) The network plot of each comparison in terms of all-cause death. (**E**) The network plot of each comparison in terms of a composite of urinary and reproductive infections. (**F**) The network plot of each comparison in terms of NT-proBNP. (**G**) The network plot of each comparison in terms of E/e’ (**H**) The network plot of each comparison in terms of LVMi. (**I**) The network plot of each comparison in terms of LVEDV. (**J**) The network plot of each comparison in terms of LVESV. (**K**) The network plot of each comparison in terms of LVEF.

The network plot presented in Figure 5 indicates that compared to placebo, sotagliflozin, dapagliflozin, and empagliflozin did not significantly reduce a composite of hospitalization for HF and CV death, individual hospitalizations for HF and CV death, or all-cause mortality, as detailed in Table 6. No significant differences were observed between the different SGLT2i treatments in these outcomes. The ranking based on SUCRA values is shown in Table 7.

**Table 6:** Comparison of the efficacy and safety of different SGLT2i in treating HFpEF [RR and mean difference (95% CI)].

Hospitalization for HF				
Empagliflozin				
0.81 (0.35, 1.86)	Dapagliflozin			
0.79 (0.29, 2.14)	0.98 (0.32, 2.96)	Sotagliflozin		
0.60 (0.03, 10.72)	0.75 (0.04, 13.84)	0.77 (0.04, 14.44)	Canagliflozin	
0.57 (0.32, 1.02)	0.71 (0.34, 1.51)	0.73 (0.32, 1.66)	0.95 (0.06, 15.95)	Placebo
A composite of hospitalization for HF and CV death				
Sotagliflozin				
0.94 (0.28, 3.13)	Dapagliflozin			
0.69 (0.23, 2.12)	0.74 (0.27, 2.01)	Empagliflozin		
0.51 (0.21, 1.26)	0.55 (0.25, 1.22)	0.74 (0.38, 1.42)	Placebo	
CV death				
Dapagliflozin				
0.87 (0.23, 3.25)	Sotagliflozin			
0.82 (0.28, 2.43)	0.95 (0.26, 3.40)	Empagliflozin		
0.61 (0.26, 1.42)	0.70 (0.25, 1.91)	0.74 (0.34, 1.61)	Placebo	
All-cause death				
Empagliflozin				
0.97 (0.79, 1.19)	Dapagliflozin			
0.90 (0.53, 1.53)	0.93 (0.55, 1.58)	Sotagliflozin		
0.91 (0.78, 1.06)	0.94 (0.82, 1.08)	1.01 (0.61, 1.69)	Placebo	
A composite of urinary and reproductive infections				
Canagliflozin				
0.24 (0.03, 2.04)	Placebo			
0.17 (0.02, 1.50)	**0.73 (0.61, 0.87)**	Empagliflozin		
0.12 (0.01, 1.20)	0.52 (0.25, 1.08)	0.72 (0.34, 1.52)	Dapagliflozin	
**0.09 (0.01, 0.86)**	**0.36 (0.15, 0.84)**	0.49 (0.21, 1.18)	0.69 (0.22, 2.12)	Sotagliflozin
E/e’				
Dapagliflozin				
−1.36 (−4.33, 1.60)	Canagliflozin			
−**1.46 (−2.19, −0.74)**	−0.10 (−2.98, 2.78)	Placebo		
−1.86 (−4.16, 0.43)	−0.50 (−4.11, 3.10)	−0.40 (−2.57, 1.77)	Ipragliflozin	
LVEF				
Dapagliflozin				
0.69 (−7.36, 8.73)	Ipragliflozin			
1.59 (−6.27, 9.44)	0.90 (−9.81, 11.61)	Canagliflozin		
**2.79 (0.37, 5.20)**	2.10 (−5.58, 9.78)	1.20 (−6.27, 8.68)	Placebo	
NT-proBNP				
Dapagliflozin				
−144.86 (−646.35, 356.64)	Canagliflozin			
−**272.79 (−469.26, −76.32)**	−127.93 (−608.67, 352.80)	Placebo		
LVMi				
Ipragliflozin				
−14.77 (−42.20, 12.67)	Dapagliflozin			
−19.10 (−46.31, 8.11)	**−4.33 (−7.80, −0.86)**	Placebo		
LVESV				
Dapagliflozin				
−0.32 (−21.43, 20.79)	Ipragliflozin			
−6.52 (−15.12, 2.07)	−6.20 (−25.48, 13.08)	Placebo		
LVEDV				
Ipragliflozin				
3.54 (−29.68, 36.76)	Dapagliflozin			
−6.70 (−37.71, 24.31)	−10.24 (−22.16, 1.68)	Placebo		

In bold: values of statistical significance (*P *< 0.05).

**Table 7:** Ranking probability of the efficacy of four in patients with HFpEF.

Ranking	A composite of hospitalization for HF and CV death	Hospitalization for HF	CV death	All-cause death	A composite of urinary and reproductive infections	NT-ProBNP	E/e’	LVMi	LVEDV	LVESV	LVEF
1	Sotagliflozin (73%)	Empagliflozin (74%)	Dapagliflozin (70%)	Empagliflozin (71%)	Canagliflozin (96%)	Dapagliflozin (87%)	Dapagliflozin (99%)	Ipragliflozin (87%)	Dapagliflozin (77%)	Dapagliflozin (71%)	Dapagliflozin (77%)
2	Dapagliflozin (70%)	Dapagliflozin (55%)	Sotagliflozin (57%)	Dapagliflozin (59%)	Placebo (75%)	Canagliflozin (48%)	Canagliflozin (37%)	Dapagliflozin (55%)	Ipragliflozin (54%)	Ipragliflozin (61%)	ipragliflozin (53%)
3	Empagliflozin (44%)	Sotagliflozin (53%)	Empagliflozin (53%)	Sotagliflozin (41%)	Empagliflozin (44%)	Placebo (14%)	Placebo (36%)	Placebo (5%)	Placebo (19%)	Placebo (16%)	Canagliflozin (43%)
4	Placebo (11%)	Canagliflozin (43%)	Placebo (19%)	Placebo (27%)	Dapagliflozin (26%)		Ipragliflozin (26%)				Placebo (24%)
5		Placebo (23%)			Sotagliflozin (8%)						

In terms of safety for patients with HFpEF, empagliflozin [RR = 0.73, CI (0.61–0.87)] and sotagliflozin [RR = 0.36, CI (0.15–0.84)] increased the risk of urinary and reproductive tract infections compared to placebo, while canagliflozin [RR = 0.09, CI (0.01–0.86)] presented a lower risk than sotagliflozin. The ranking based on SUCRA values for safety is as follows: canagliflozin (96%), placebo (75%), empagliflozin (44%), dapagliflozin (26%), and sotagliflozin (8%).

Compared to placebo, dapagliflozin [MD = −272.79, CI (−469.26 to −76.32)] showed significant differences in reducing NT-proBNP levels, while canagliflozin showed no difference. There were no significant differences among the different SGLT2i treatments. The ranking based on SUCRA values is as follows: dapagliflozin (87%), canagliflozin (48%), and placebo (14%). In addition, compared to placebo, dapagliflozin improved E/e' [MD = −1.46, CI (−2.19 to −0.74)], LVMi [MD = −4.33, CI (−7.80 to −0.86)], and LVEF [MD = 2.79, CI (0.37–5.20)] in terms of adverse cardiac remodeling. No differences were noted for canagliflozin and ipragliflozin or among different SGLT2i. There was no statistical difference in improving LVESV and LVEDV compared to placebo and among the different SGLT2i treatments. The ranking based on SUCRA values is detailed in Table 7.

#### Consistency and small sample study effect

**Figure 6:**
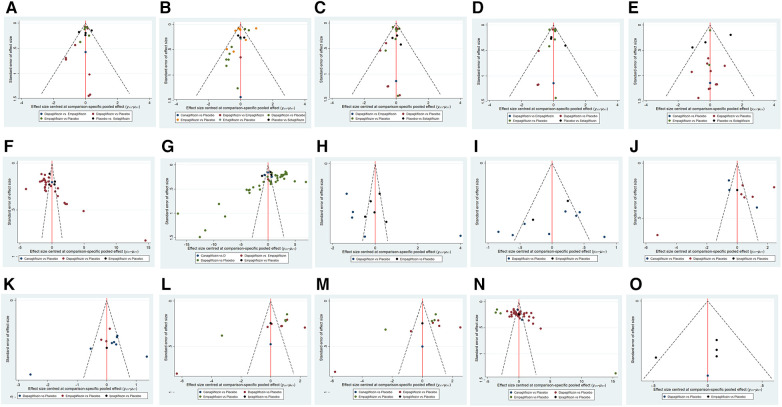
Funnel plot of pairwise comparison among each SGLT2i treatment. (**A**) The funnel plot of a composite of hospitalization for HF and CV death. (**B**) The funnel plot of hospitalization for HF. (**C**) The funnel plot of CV death. (**D**) The funnel plot of all-cause death. (**E**) The funnel plot of a composite of urinary and reproductive infections. (**F**) The funnel plot of 6 min walk distance. (**G**) The funnel plot of NT-proBNP. (**H**) The funnel plot of KCCQ. (**I**) The funnel plot of LAVi. (**J**) The funnel plot of E/e’. (**K**) The funnel plot of LVMi. (**L**) The funnel plot of LVEDV. (**M**) The funnel plot of LVESV. (**N**) The funnel plot of LVEF. (**O**) The funnel plot of HCT.

Comparison-corrected funnel plots were utilized to assess publication bias in the study, focusing on a range of outcome indicators such as a composite of hospitalization for HF and CV death, hospitalization for HF, CV death, all-cause death, urinary and reproductive infections, 6MWT, NT-ProBNP, KCCQ, LAVi, E/e', LVMi, LVEDV, LVESV, LVEF, and HCT. The network funnel plot revealed the presence of small sample effects in the comparison between dapagliflozin and placebo for a composite of hospitalization for HF and CV death (Figure 6A), a composite of urinary and reproductive infections (Figure 6E), CV death (Figure 6C), 6MWT and NT-ProBNP (Figures 6F,G), LVMi (Figure 6K), LVESV (Figure 6M), and LVEDV (Figure 6L). The comparison between canagliflozin and placebo showed a small sample effect in hospitalization for HF (Figure 6B), while the comparison between empagliflozin and placebo indicated small sample effects in CV death (Figure 6C), LVESV (Figure 6M), LVEF (Figure 6N), and all-cause death (Figure 6D).

## Discussion

This review analyzed 77 RCT involving 43,561 patients using Bayesian network meta-analysis for a comprehensive evaluation. The study encompassed more than 10 outcome indicators, including a composite of hospitalization for HF and CV death, hospitalization for HF and CV death, a composite of urinary and reproductive effects, and an assessment of the cardiac structure. Subgroup analysis was performed based on the ejection fraction of HF. Although the efficacy of SGLT2i varies slightly with different LVEF baselines of patients, it may be beneficial in patients with HF regardless of LVEF baseline. Compared with the placebo, SGLT2i demonstrated a significant advantage in reducing a composite of hospitalization for HF and CV death, hospitalization for HF and CV death, and KCQQ scores while showing no significant impact on reducing all-cause mortality. Indirect comparisons between different SGLT2i suggest improvements in a composite of hospitalization for HF and CV death, hospitalization for HF, and CV death. Sotagliflozin outperformed empagliflozin and dapagliflozin in reducing hospitalization for HF and CV death. However, there is no difference between empagliflozin and dapagliflozin. Nevertheless, given the limited research on sotagliflozin, further investigation is warranted.

Regarding the safety profile in total HF patients, SGLT2i are associated with an increased risk of urinary and reproductive system infections, with dapagliflozin showing the highest risk among them. However, there is no distinction between various types of SGLT2i. A previous meta-analysis (83) showed that, except for dapagliflozin, SGLT2i did not increase incidences of urinary and reproductive system infections, which is consistent with our findings. Moreover, the US Food and Drug Administration has included this potential side effect in its list of adverse reactions. HCT was utilized as a reference indicator to assess low blood volume. The meta-analysis demonstrated that SGLT2i resulted in a rise in HCT relative to placebo, implying an elevated hypotension hazard for SGLT2i. The primary mechanism of action of SGLT2i involves the inhibition of the SGLT2 transporter, predominantly located in the S1 segment of the proximal tubules (84), increasing the excretion of glucose in the urine. Nevertheless, inhibiting SGLT2i also diminishes sodium reabsorption in the proximal tubules, potentially increasing sodium excretion. Previous studies (85, 86) reported a correlation between the administration of SGLT2i and a reduction of body weight and blood pressure.

The network meta-analysis results indicated that dapagliflozin and empagliflozin significantly improved NT-proBNP and 6MWT. However, no statistically significant difference was observed among different SGLT2i. While SGLT2i have shown promise in treating HF, it is crucial to determine whether they directly influence the heart's structural function. Therefore, we collected relevant indicators, such as LAVi, E/e', LVMi, and LVEDV, to systematically evaluate the changes in cardiac structure in HF patients treated with SGLT2i. The results showed that SGLT2i significantly reduced LVMi, LVEDV, LAVi, and LVESV and increased LVEF, reflecting significant benefits in improving cardiac systolic and diastolic function. Cardiac anatomy and functional parameters are vital in predicting the prognosis and quality of life in HF patients. Animal studies conducted on T2DM models revealed the positive effects of SGLT2i on left ventricular hypertrophy and dilation (87, 88), as well as cardiac systolic and diastolic function. LAVi and E/e' are predictive factors (89) used to evaluate cardiac diastolic function. Our research revealed that SGLT2i did not reduce E/e' in HFrEF patients. Nonetheless, subgroup analysis indicated SGLT2i could enhance the E/e' and LAVi of patients with HFpEF, potentially due to differing mechanisms between HFpEF and HFrEF.

According to the grading of HFpEF by the European and American Heart Association, this study conducted subgroup analysis based on ejection fraction. The HFpEF (EF ≥ 50%) group did not show significant differences in reducing a composite of hospitalization for HF and CV death, hospitalization for HF, CV death, and all-cause death. No significant differences were observed between different SGLT2i. However, some meta-analyses have shown that (90–92) SGLT2i can reduce a composite of HF and CV death hospitalizations. However, the previous study defined HFpEF as EF greater than 40%, which differs from our study. Therefore, our study should be more convincing. Regarding the safety of HFpEF patients, SGLT2i also present risks of urinary and reproductive infections, with empagliflozin and sotagliflozin being notable culprits. Canagliflozin has demonstrated higher safety compared to sotagliflozin in this aspect. In terms of improving ventricular remodeling, compared to placebo, SGLT2i have shown improvement in LAVi, E/e', LVEF, and LVMi. However, no significant differences were observed in LVESV and LVEDV, and there was no difference between different SGLT2i. The mechanism of HFpEF remains unclear, and left ventricular diastolic dysfunction is considered the main pathophysiological mechanism underlying the occurrence of HFpEF (93). Our research also confirmed that SGLT2i can improve the diastolic function of HFpEF patients. Typically, remodeling the structure of the patient's heart can significantly enhance their prognosis and quality of life. Unfortunately, there has been limited research on the quality of life scores of HFpEF patients; thus, this outcome measure was not included in our analysis.

In the subgroup analysis of HFrEF (EF <50%), the network meta-analysis results revealed significant effects of SGLT2i in reducing hospitalization for HF, CV death, all-cause death, NT-ProBNP, and 6MWT. Interestingly, this finding contrasts with the statistical results obtained before conducting the subgroup analysis, indicating the importance of evaluating the contribution of SGLT2i to HF based on ejection fraction. Furthermore, SGLT2i showed significant advantages in improving all-cause death. The indirect comparison revealed no statistical difference between different SGLT2i. Regarding the safety of HFrEF, dapagliflozin significantly increased the risk of a composite of urinary and reproductive infections compared to placebo. Additionally, our analysis revealed that SGLT2i could enhance KCCQ scores in HFrEF patients. Regarding ventricular remodeling, our study revealed that SGLT2i reduced LVMi, LVESV, and LVEDV and increased LVEF. These findings suggest that SGLT2i can enhance diastolic and systolic function in patients with HFrEF, thereby potentially augmenting the prognostic outcomes for these patients. The therapeutic effect of SGLT2i on cardiac structural remodeling was found to be significantly better in HFrEF patients compared to HFpEF patients, with SGLT2i demonstrating superiority in improving cardiac diastolic function in HFpEF patients. Consistent with our findings, a previous meta-analysis (94) showed that empagliflozin had a more significant effect in improving cardiac structure.

This study presents several limitations. ① This study mainly focuses on empagliflozin and dapagliflozin, with relatively little research available on canagliflozin, sotagliflozin, ipragliflozin, and ertugliflozin. Future research should explore these alternative SGLT2i to provide a more comprehensive understanding of their efficacy and safety profiles. ② Currently, there is only one direct comparison between dapagliflozin and empagliflozin available in current literature, leading to an indirect evaluation of the efficacy and safety of canagliflozin, sotagliflozin, ipragliflozin, and ertugliflozin in treating HF patients. Consequently, a potential bias exists between the reported results and the actual drug performances, underscoring the need for further direct-controlled trials to validate their efficacy and safety profiles. ③ There are variations in baseline characteristics such as gender, age, race, and chronic medical conditions among the included studies, potentially resulting in clinical heterogeneity. ④ Variations in follow-up durations between the six SGLT2i drug studies and within individual studies for each drug could introduce bias into the study results. ⑤ The limited number of studies on HF with HFpEF (EF ≥ 50%) underscores the necessity for more research to substantiate the relevant findings.

## Conclusion

In summary, SGLT2i can significantly improve the prognosis of all patients with HF despite the associated increased risk of urinary and reproductive infections. Overall, HF patients benefit from enhanced cardiac remodeling, with those with HFrEF experiencing the most substantial benefits. Indirect comparisons between different SGLT2i revealed no significant differences in HFrEF. Among the six types of SGLT2i, sotagliflozin demonstrated superiority over empagliflozin and dapagliflozin in reducing hospitalization for HF and cardiovascular death in total HF. Canagliflozin exhibited higher safety than sotagliflozin regarding urinary and reproductive infections in patients with HFpEF. Overall, SGLT2i showed better efficacy in patients with HFrEF than those with HFpEF.

## Data Availability

The original contributions presented in the study are included in the article/supplementary material, further inquiries can be directed to the corresponding author.
