# Supplementary material for: Effects of different sodium–glucose cotransporter 2 inhibitors in heart failure with reduced or preserved ejection fraction: a network meta-analysis
Source: Front Cardiovasc Med. 2024 May 23;11:1379765. doi: 10.3389/fcvm.2024.1379765 (PMC11153861; doi:10.3389/fcvm.2024.1379765)
Supplement: Supplementary file 1 [file Datasheet1.docx]

**Table.S1 Search strategy**

| Electronic database | Search strategy |
| --- | --- |
| PubMed (NCBI) | 1# (Sodium Glucose Transporter 2 Inhibitors) OR (SGLT-2 Inhibitors) OR (SGLT 2 Inhibitors) OR (SGLT2 Inhibitors) OR (Sodium-Glucose Transporter 2 Inhibitor) OR (Sodium Glucose Transporter 2 Inhibitor) OR (SGLT2 Inhibitor) OR (Inhibitor, SGLT2) OR (Gliflozins) OR (Gliflozin) OR (SGLT-2 Inhibitor) OR (Inhibitor, SGLT-2) OR (SGLT 2 Inhibitor) OR (canagliflozin) OR (dapagliflozin) OR (empagliflozin) OR (ipragliflozin) OR (Luseogliflozin) OR (Sotagliflozin) OR (Remogliflozin) OR (Sergliflozin) OR (Tofogliflozin) OR (ertugliflozin)  2# Heart failure[Title/Abstract]  3# (Heart Decompensation[Title/Abstract]) OR (Decompensation, Heart[Title/Abstract]) OR (Heart Failure, Right-Sided[Title/Abstract]) OR (Heart Failure, Right Sided[Title/Abstract]) OR (Right-Sided Heart Failure[Title/Abstract]) OR (Right Sided Heart Failure[Title/Abstract]) OR (Myocardial Failure[Title/Abstract]) OR (Congestive Heart Failure[Title/Abstract]) OR (Heart Failure, Congestive[Title/Abstract]) OR (Heart Failure, Left-Sided[Title/Abstract]) OR (Heart Failure, Left Sided[Title/Abstract]) OR (Left-Sided Heart Failure[Title/Abstract]) OR (Left Sided Heart Failure[Title/Abstract])  4# (Heart failure[Title/Abstract]) OR ((Heart Decompensation[Title/Abstract]) OR (Decompensation, Heart[Title/Abstract]) OR (Heart Failure, Right-Sided[Title/Abstract]) OR (Heart Failure, Right Sided[Title/Abstract]) OR (Right-Sided Heart Failure[Title/Abstract]) OR (Right Sided Heart Failure[Title/Abstract]) OR (Myocardial Failure[Title/Abstract]) OR (Congestive Heart Failure[Title/Abstract]) OR (Heart Failure, Congestive[Title/Abstract]) OR (Heart Failure, Left-Sided[Title/Abstract]) OR (Heart Failure, Left Sided[Title/Abstract]) OR (Left-Sided Heart Failure[Title/Abstract]) OR (Left Sided Heart Failure[Title/Abstract]))  5# ((((Random*[Title/Abstract]) ) OR (RCT[Title/Abstract])) OR (RCTs[Title/Abstract])) OR (Randomized Controlled Trial[Publication Type])  6# ((Sodium Glucose Transporter 2 Inhibitors) OR (SGLT-2 Inhibitors) OR (SGLT 2 Inhibitors) OR (SGLT2 Inhibitors) OR (Sodium-Glucose Transporter 2 Inhibitor) OR (Sodium Glucose Transporter 2 Inhibitor) OR (SGLT2 Inhibitor) OR (Inhibitor, SGLT2) OR (Gliflozins) OR (Gliflozin) OR (SGLT-2 Inhibitor) OR (Inhibitor, SGLT-2) OR (SGLT 2 Inhibitor) OR (canagliflozin) OR (dapagliflozin) OR (empagliflozin) OR (ipragliflozin) OR (Luseogliflozin) OR (Sotagliflozin) OR (Remogliflozin) OR (Sergliflozin) OR (Tofogliflozin) OR (ertugliflozin)) AND ((Heart failure[Title/Abstract]) OR ((Heart Decompensation[Title/Abstract]) OR (Decompensation, Heart[Title/Abstract]) OR (Heart Failure, Right-Sided[Title/Abstract]) OR (Heart Failure, Right Sided[Title/Abstract]) OR (Right-Sided Heart Failure[Title/Abstract]) OR (Right Sided Heart Failure[Title/Abstract]) OR (Myocardial Failure[Title/Abstract]) OR (Congestive Heart Failure[Title/Abstract]) OR (Heart Failure, Congestive[Title/Abstract]) OR (Heart Failure, Left-Sided[Title/Abstract]) OR (Heart Failure, Left Sided[Title/Abstract]) OR (Left-Sided Heart Failure[Title/Abstract]) OR (Left Sided Heart Failure[Title/Abstract])))  7# (((((Random*[Title/Abstract]) ) OR (RCT[Title/Abstract])) OR (RCTs[Title/Abstract])) OR (Randomized Controlled Trial[Publication Type])) AND (((Sodium Glucose Transporter 2 Inhibitors) OR (SGLT-2 Inhibitors) OR (SGLT 2 Inhibitors) OR (SGLT2 Inhibitors) OR (Sodium-Glucose Transporter 2 Inhibitor) OR (Sodium Glucose Transporter 2 Inhibitor) OR (SGLT2 Inhibitor) OR (Inhibitor, SGLT2) OR (Gliflozins) OR (Gliflozin) OR (SGLT-2 Inhibitor) OR (Inhibitor, SGLT-2) OR (SGLT 2 Inhibitor) OR (canagliflozin) OR (dapagliflozin) OR (empagliflozin) OR (ipragliflozin) OR (Luseogliflozin) OR (Sotagliflozin) OR (Remogliflozin) OR (Sergliflozin) OR (Tofogliflozin) OR (ertugliflozin)) AND ((Heart failure[Title/Abstract]) OR ((Heart Decompensation[Title/Abstract]) OR (Decompensation, Heart[Title/Abstract]) OR (Heart Failure, Right-Sided[Title/Abstract]) OR (Heart Failure, Right Sided[Title/Abstract]) OR (Right-Sided Heart Failure[Title/Abstract]) OR (Right Sided Heart Failure[Title/Abstract]) OR (Myocardial Failure[Title/Abstract]) OR (Congestive Heart Failure[Title/Abstract]) OR (Heart Failure, Congestive[Title/Abstract]) OR (Heart Failure, Left-Sided[Title/Abstract]) OR (Heart Failure, Left Sided[Title/Abstract]) OR (Left-Sided Heart Failure[Title/Abstract]) OR (Left Sided Heart Failure[Title/Abstract])))) |
| Embase | #1. 'heart failure'/exp OR 'heart failure' OR  'cardiac failure'/exp OR 'cardiac failure' OR  'heart decompensation'/exp OR 'heart  decompensation' OR 'decompensation, heart'/exp OR  'decompensation, heart' OR 'heart failure,  right-sided' OR 'heart failure, right sided' OR  'right-sided heart failure' OR 'right sided heart  failure'/exp OR 'right sided heart failure' OR  'myocardial failure'/exp OR 'myocardial failure'  OR 'congestive heart failure'/exp OR 'congestive  heart failure' OR 'heart failure, congestive'/exp  OR 'heart failure, congestive' OR 'heart failure,  left-sided' OR 'heart failure, left sided' OR  'left-sided heart failure'  #2. 'randomized controlled trial' OR 'random*' OR  'rct' OR 'rcts'  #3. 'sodium glucose transporter 2 inhibitors' OR  'sglt-2 inhibitors' OR 'sglt 2 inhibitors' OR  'sglt2 inhibitors' OR 'sodium-glucose transporter  2 inhibitor' OR 'sodium glucose transporter 2  inhibitor' OR 'sglt2 inhibitor' OR 'inhibitor,  sglt2' OR 'gliflozins' OR 'gliflozin' OR 'sglt-2  inhibitor' OR 'inhibitor, sglt-2' OR 'sglt 2  inhibitor' OR 'canagliflozin' OR 'dapagliflozin'  OR 'empagliflozin' OR 'ipragliflozin' OR  'luseogliflozin' OR 'sotagliflozin' OR  'remogliflozin' OR 'sergliflozin' OR  'tofogliflozin' OR 'ertugliflozin'  #4. #1 AND #3  #5. #2 AND #4 |
| Cochrane | #1. 'heart failure'/exp OR 'heart failure' OR  'cardiac failure'/exp OR 'cardiac failure' OR  'heart decompensation'/exp OR 'heart  decompensation' OR 'decompensation, heart'/exp OR  'decompensation, heart' OR 'heart failure,  right-sided' OR 'heart failure, right sided' OR  'right-sided heart failure' OR 'right sided heart  failure'/exp OR 'right sided heart failure' OR  'myocardial failure'/exp OR 'myocardial failure'  OR 'congestive heart failure'/exp OR 'congestive  heart failure' OR 'heart failure, congestive'/exp  OR 'heart failure, congestive' OR 'heart failure,  left-sided' OR 'heart failure, left sided' OR  'left-sided heart failure'  #2. 'randomized controlled trial' OR 'random*' OR  'rct' OR 'rcts'  #3. 'sodium glucose transporter 2 inhibitors' OR  'sglt-2 inhibitors' OR 'sglt 2 inhibitors' OR  'sglt2 inhibitors' OR 'sodium-glucose transporter  2 inhibitor' OR 'sodium glucose transporter 2  inhibitor' OR 'sglt2 inhibitor' OR 'inhibitor,  sglt2' OR 'gliflozins' OR 'gliflozin' OR 'sglt-2  inhibitor' OR 'inhibitor, sglt-2' OR 'sglt 2  inhibitor' OR 'canagliflozin' OR 'dapagliflozin'  OR 'empagliflozin' OR 'ipragliflozin' OR  'luseogliflozin' OR 'sotagliflozin' OR  'remogliflozin' OR 'sergliflozin' OR  'tofogliflozin' OR 'ertugliflozin'  #4. #1 AND #3  #5. #2 AND #4 |
| Sinomed | (心力衰竭 OR 心脏代偿失调 OR 心脏衰竭 OR 右侧心脏衰竭 OR 充血性心力衰竭) AND (达格列净 OR 恩格列净 OR 卡格列净 OR 异格列净 OR 厄格列净 OR 柳格列净OR钠-葡萄糖协同转运蛋白2抑制剂 OR SGLT-2 Inhibitors) |
| WOS | #1 TS=(Sodium Glucose Transporter 2 Inhibitors OR SGLT-2 Inhibitors OR SGLT 2 Inhibitors OR SGLT2 Inhibitors OR Sodium-Glucose Transporter 2 Inhibitor OR Sodium Glucose Transporter 2 Inhibitor OR SGLT2 Inhibitor OR Inhibitor, SGLT2 OR Gliflozins OR Gliflozin OR SGLT-2 Inhibitor OR Inhibitor, SGLT-2 OR SGLT 2 Inhibitor OR canagliflozin OR dapagliflozin OR empagliflozin OR ipragliflozinOR Luseogliflozin OR Sotagliflozin OR Remogliflozin OR Sergliflozin OR Tofogliflozin OR ertugliflozin)  #2 TS=((Randomized Controlled Trial OR Random* OR RCT OR RCTs))  #3 TS=(Cardiac Failure OR Heart Decompensation OR Decompensation, Heart OR Heart Failure, Right-Sided OR Heart Failure, Right Sided OR Right-Sided Heart Failure OR Right Sided Heart Failure OR Myocardial Failure OR Congestive Heart Failure OR Heart Failure, Congestive OR Heart Failure, Left-Sided OR Heart Failure, Left Sided OR Left-Sided Heart Failure OR Left Sided Heart Failure)  #4 TS=(Heart failure)  #5 #4 OR #3  #6 #1 AND #5  #7 #2 AND #6 |
| WanFangdata | 主题:(达格列净 OR 恩格列净 OR 卡格列净 OR 异格列净 OR 厄格列净 OR 柳格列净 OR 钠-葡萄糖协同转运蛋白2抑制剂 OR SGLT-2 Inhibitors) and 主题:(心力衰竭 OR 心脏代偿失调 OR 心脏衰竭 OR 右侧心脏衰竭 OR 充血性心力衰竭) |
| cochrance | #1 (Heart failure):ab,ti,kw OR (Cardiac Failure):ab,ti,kw OR (Heart Decompensation):ab,ti,kw OR (Decompensation, Heart):ab,ti,kw OR (Heart Failure, Right-Sided):ab,ti,kw OR (Heart Failure, Right Sided):ab,ti,kw OR (Right-Sided Heart Failure):ab,ti,kw OR (Right Sided Heart Failure):ab,ti,kw OR (Myocardial Failure):ab,ti,kw OR (Congestive Heart Failure):ab,ti,kw OR (Heart Failure, Congestive):ab,ti,kw OR (Heart Failure, Left-Sided):ab,ti,kw OR (Heart Failure, Left Sided):ab,ti,kw OR (Left-Sided Heart Failure):ab,ti,kw OR (Left Sided Heart Failure):ab,ti,kw  #2 (Sodium Glucose Transporter 2 Inhibitors):ab,ti,kw OR (SGLT-2 Inhibitors):ab,ti,kw OR (SGLT 2 Inhibitors):ab,ti,kw OR (SGLT2 Inhibitors):ab,ti,kw OR (Sodium-Glucose Transporter 2 Inhibitor):ab,ti,kw OR (Sodium Glucose Transporter 2 Inhibitor):ab,ti,kw OR (SGLT2 Inhibitor):ab,ti,kw OR (Inhibitor, SGLT2):ab,ti,kw OR (Gliflozins):ab,ti,kw OR (Gliflozin):ab,ti,kw OR (SGLT-2 Inhibitor):ab,ti,kw OR (Inhibitor, SGLT-2):ab,ti,kw OR (SGLT 2 Inhibitor):ab,ti,kw OR (canagliflozin):ab,ti,kw OR (dapagliflozin):ab,ti,kw OR (empagliflozin):ab,ti,kw OR (ipragliflozin):ab,ti,kw OR (Luseogliflozin):ab,ti,kw OR (Sotagliflozin):ab,ti,kw OR (Remogliflozin):ab,ti,kw OR (Sergliflozin):ab,ti,kw OR (Tofogliflozin):ab,ti,kw OR (ertugliflozin):ab,ti,kw  #3 (Randomized Controlled Trial):ab,ti,kw OR (Random*):ab,ti,kw OR (RCT):ab,ti,kw OR (RCTs):ab,ti,kw  #4 #1 AND #2  #5 #3 AND #4 |
| VIP | (((((((((题名或关键词=心力衰竭 OR 题名或关键词=慢性心功能不全) OR 题名或关键词=慢性心脏功能衰竭) OR 题名或关键词=慢性心力衰竭) OR 题名或关键词=慢性心衰) OR 题名或关键词=心力衰竭) AND (((((((题名或关键词=达格列净 OR 题名或关键词=恩格列净) OR 题名或关键词=卡格列净) OR 题名或关键词=异格列净) OR 题名或关键词=厄格列净) OR 题名或关键词=柳格列净) OR 题名或关键词=钠葡萄糖协同转运蛋白2抑制剂) OR (题名或关键词=SGLT AND ( NOT 题名或关键词=2 Inhibitors))))) AND (((摘要=随机对照 OR 摘要=随机) OR 摘要=随机分配) OR 摘要=RCT))) |
| CNKI | 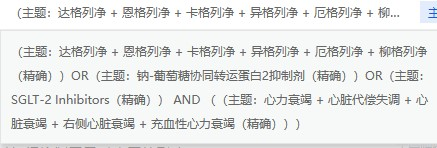 |
