# Supplementary figures and images for: Effects of different sodium–glucose cotransporter 2 inhibitors in heart failure with reduced or preserved ejection fraction: a network meta-analysis
Source: Front Cardiovasc Med. 2024 May 23;11:1379765. doi: 10.3389/fcvm.2024.1379765 (PMC11153861; doi:10.3389/fcvm.2024.1379765)

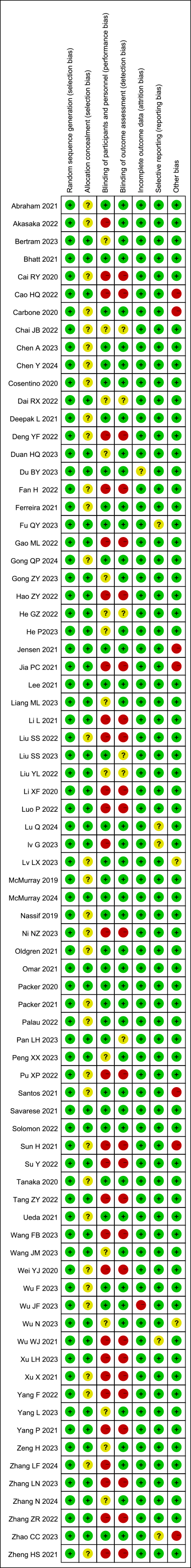

Supplement: Supplementary file 2 [file Image1.tif]
